# Supplementary material for: Coincidence of cholinergic pauses, dopaminergic activation and depolarisation of spiny projection neurons drives synaptic plasticity in the striatum
Source: Nat Commun. 2022 Mar 11;13:1296. doi: 10.1038/s41467-022-28950-0 (PMC8917208; doi:10.1038/s41467-022-28950-0)
Supplement: Supplementary file 1 — Supplementary Information [file 41467_2022_28950_MOESM1_ESM.pdf]

Supplementary information

Coincidence of cholinergic pauses, dopaminergic activation and depolarisation of spiny projection neurons drives synaptic plasticity in the striatum

John N. J. Reynolds[1*†], Riccardo Avvisati[2], Paul D. Dodson[2], Simon D. Fisher[1], Manfred J. Oswald[1], Jeffery R. Wickens[1,3], & Yan-Feng Zhang[1,4*†]

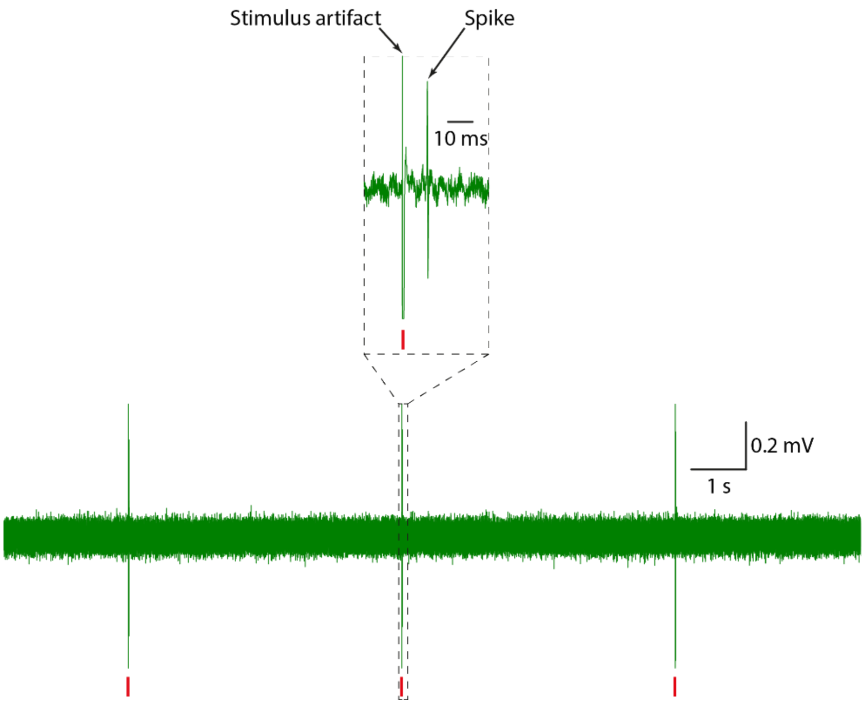

Supplementary Fig. 1. Extracellular recording of a SPN. A SPN with no spontaneous spiking activity fired action potentials only following cortical stimulation (red lines).

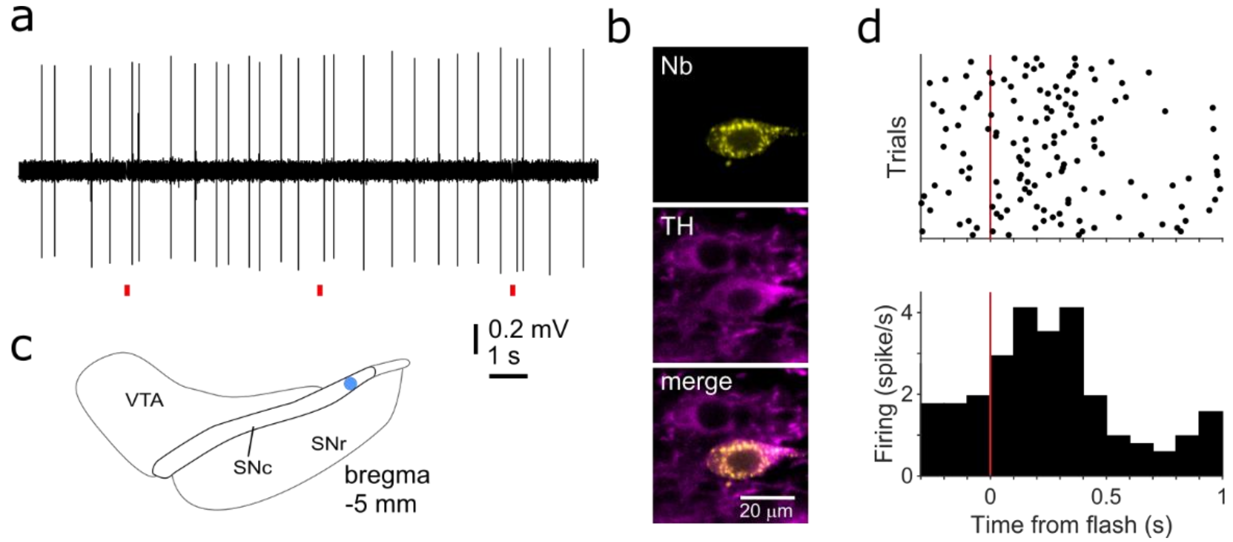

Supplementary Fig. 2. Phasic spike activity of a dopamine neuron induced by a light flash after bicuculline injection into the SC. **a** Extracellular single-unit *in vivo* recording from an identified midbrain dopamine neuron in the substantia nigra pars compacta (SNc) (**b-c**). Following bicuculline injection into the SC, a light flash to the contralateral eye (red dash in a) caused the neuron to increase its firing rate (**a**, **d**) as described by Dommett et al. (2005). **b** Following recording, the neuron was juxtacellularly labelled with neurobiotin (Nb) to determine its location (a representative example from two neurons) (**c**) and tested for immunoreactivity to tyrosine hydroxylase (TH) to confirm its neurochemical identity.
