# Supplementary material for: Coincidence of cholinergic pauses, dopaminergic activation and depolarisation of spiny projection neurons drives synaptic plasticity in the striatum
Source: Nat Commun. 2022 Mar 11;13:1296. doi: 10.1038/s41467-022-28950-0 (PMC8917208; doi:10.1038/s41467-022-28950-0)
Supplement: Supplementary file 3 — Reporting Summary [file 41467_2022_28950_MOESM3_ESM.pdf]

nature portfolio

Corresponding author(s): Yan-Feng Zhang; John NJ Reynolds

Last updated by author(s): Jan 12, 2022

Reporting Summary

Nature Portfolio wishes to improve the reproducibility of the work that we publish. This form provides structure for consistency and transparency in reporting. For further information on Nature Portfolio policies, see our Editorial Policies and the Editorial Policy Checklist.

Statistics

For all statistical analyses, confirm that the following items are present in the figure legend, table legend, main text, or Methods section.

n/a	Confirmed	
☐	☒	The exact sample size (*n*) for each experimental group/condition, given as a discrete number and unit of measurement
☐	☒	A statement on whether measurements were taken from distinct samples or whether the same sample was measured repeatedly
☐	☒	The statistical test(s) used AND whether they are one- or two-sided<br>*Only common tests should be described solely by name; describe more complex techniques in the Methods section.*
☒	☐	A description of all covariates tested
☐	☒	A description of any assumptions or corrections, such as tests of normality and adjustment for multiple comparisons
☐	☒	A full description of the statistical parameters including central tendency (e.g. means) or other basic estimates (e.g. regression coefficient) AND variation (e.g. standard deviation) or associated estimates of uncertainty (e.g. confidence intervals)
☐	☒	For null hypothesis testing, the test statistic (e.g. *F*, *t*, *r*) with confidence intervals, effect sizes, degrees of freedom and *P* value noted<br>*Give P values as exact values whenever suitable.*
☒	☐	For Bayesian analysis, information on the choice of priors and Markov chain Monte Carlo settings
☒	☐	For hierarchical and complex designs, identification of the appropriate level for tests and full reporting of outcomes
☒	☐	Estimates of effect sizes (e.g. Cohen's *d*, Pearson's *r*), indicating how they were calculated

Our web collection on statistics for biologists contains articles on many of the points above.

Software and code

Policy information about availability of computer code

Data collection	Extracellular recordings were acquired with SPIKE2 software (v6 or v7, CED). Intracellular recordings were acquired with pClamp 8 software (Molecular Devices).
Data analysis	Extracellular recordings were analysed with SPIKE2 software (v6 or v7, CED). Intracellular recordings were analysed with Axograph 4.9 software.

For manuscripts utilizing custom algorithms or software that are central to the research but not yet described in published literature, software must be made available to editors and reviewers. We strongly encourage code deposition in a community repository (e.g. GitHub). See the Nature Portfolio guidelines for submitting code & software for further information.

Data

Policy information about availability of data

All manuscripts must include a data availability statement. This statement should provide the following information, where applicable:

- Accession codes, unique identifiers, or web links for publicly available datasets
- A description of any restrictions on data availability
- For clinical datasets or third party data, please ensure that the statement adheres to our policy

Source data for figure 2 and 3 are available.

March 2021

Field-specific reporting

Please select the one below that is the best fit for your research. If you are not sure, read the appropriate sections before making your selection.

☒ Life sciences ☐ Behavioural & social sciences ☐ Ecological, evolutionary & environmental sciences

For a reference copy of the document with all sections, see nature.com/documents/nr-reporting-summary-flat.pdf

Life sciences study design

All studies must disclose on these points even when the disclosure is negative.

Sample size	Sample sizes in this study are listed in the main text, and corresponding figure legends. Sample sizes employed were based on previous experiments. Sample size were not pre-determined by sample size or power calculations.
Data exclusions	All the data collected for the study are included.
Replication	No replication has been attempt as the experiment itself is extremely time consuming. The two experimental paradigms replicated each others results.
Randomization	The animals are randomly grouped to ensure the randomization of the experiments.
Blinding	No blinding was used.

Reporting for specific materials, systems and methods

We require information from authors about some types of materials, experimental systems and methods used in many studies. Here, indicate whether each material, system or method listed is relevant to your study. If you are not sure if a list item applies to your research, read the appropriate section before selecting a response.

Materials & experimental systems

n/a	Involved in the study	
☐	☒	Antibodies
☒	☐	Eukaryotic cell lines
☒	☐	Palaeontology and archaeology
☐	☒	Animals and other organisms
☒	☐	Human research participants
☒	☐	Clinical data
☒	☐	Dual use research of concern

Methods

n/a	Involved in the study	
☒	☐	ChIP-seq
☒	☐	Flow cytometry
☒	☐	MRI-based neuroimaging

Antibodies

Antibodies used	VECTASTAIN ABC Kits (HRP), Cy3-conjugated streptavidin (Sigma-Aldrich GEPA43001), chicken anti-Tyrosine Hydroxylase primary (Abcam AB76442) and Brilliant Violet 421-conjugated anti-chicken secondary antibodies (Jackson ImmunoResearch 703-675-155).
Validation	N/A

Animals and other organisms

Policy information about studies involving animals; ARRIVE guidelines recommended for reporting animal research

Laboratory animals	Male Wistar rats and Long-Evan rats weighing 250 to 420 g at the time of the recording were used.
Wild animals	The study did not involve wild animals.
Field-collected samples	The study did not involve samples collected from the field.
Ethics oversight	All procedures in this study were conducted in accordance with approvals granted by the University of Otago Animal Ethics Committee or in accordance to the UK animals scientific procedures act, 1986 with approval of the University of Bristol ethics committee.

Note that full information on the approval of the study protocol must also be provided in the manuscript.
